# Acute Myeloid Leukemia Presenting as Myeloid Sarcoma with a Predisposition to the Gynecologic Tract

**DOI:** 10.1155/2019/4189275

**Published:** 2019-12-30

**Authors:** Ryan M. Kahn, Sushmita Gordhandas, Eloise Chapman-Davis, Elizabeth Margolskee, Cathleen Matrai, Amy Chadburn, Ellen Ritchie

**Affiliations:** ^1^Department of Obstetrics and Gynecology, Weill Cornell Medicine, New York, NY, USA; ^2^Department of Obstetrics and Gynecology, Division of Gynecologic Oncology, Weill Cornell Medical College-New York Presbyterian Hospital, New York, NY 10065, USA; ^3^Department of Pathology and Laboratory Medicine, Weill Cornell Medical College-New York Presbyterian Hospital, New York, NY, USA; ^4^Division of Hematology and Oncology, Weill Cornell Medical College, New York, NY, USA

## Abstract

Myeloid sarcoma (MS) is a rare, extramedullary tumor consisting of immature white blood cells of myeloid lineage. MS is usually associated with the concurrent diagnosis of acute myeloid leukemia (AML) but can also present in the absence of bone marrow disease or at relapse of AML. MS of the gynecologic tract is exceedingly rare; however, it is hypothesized that it is likely more prevalent than previously understood given postmortem findings and persistence in preserved ovarian tissue. There is minimal literature surrounding MS and extramedullary relapse with no clear guidelines. This is a case report of a 48-year-old woman with MS involving the uterine corpus, fallopian tubes, and left ovary followed by a literature review. The overall aim is to review data regarding leukemic immune evasion and sanctuary sites in order to raise awareness as this represents an important and underrecognized hematologic malignancy in an often misdiagnosed, underrecognized site.

## 1. Introduction

Myeloid sarcoma (MS) is a rare, extramedullary tumor consisting of immature white blood cells of myeloid lineage that disrupt the tissue architecture in which it invades. MS has been reported in 2-8% of patients with acute myeloid leukemia (AML) and is most commonly diagnosed concurrently with this disease [1]. Rarely, MS presents as the initial manifestation of AML or proceeds AML relapse. Myeloid sarcoma can occur in the absence of bone marrow disease, which is known as isolated or nonleukemic MS. Although MS is most commonly found in soft tissues, bone, lymph nodes, the central nervous system, testicles, and ovaries, there are rare reports of the involvement of the uterus and fallopian tubes [2]. It is widely believed that the gynecologic tract may be an important and underrecognized reservoir of leukemic blasts through immune evasion, resulting in an increased risk of disease relapse.

## 2. Case Report

**Figure 1:**
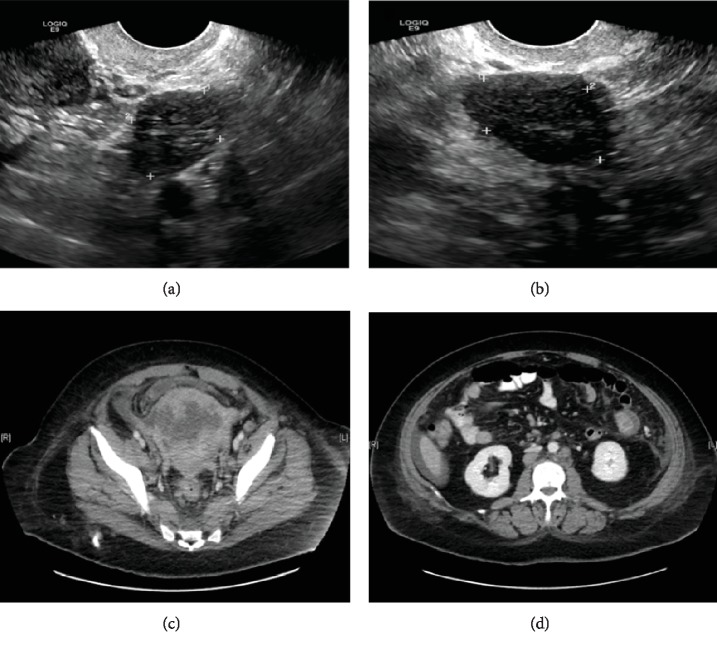
(a) The right ovary measures 3.4 × 2.4 × 2.2 cm and shows diffuse abnormal hypoechogenicity and increased vascularity. (b) The left ovary measures 2.8 × 2.2 × 2.1 cm and shows diffuse abnormal hypoechogenicity with mildly increased vascularity. (c) The cervix and uterus are enlarged and heterogeneous with multifocal hypodense and cystic foci. An irregular hypodense lesion in the anterior uterine body measuring 5.1 × 8.0 × 4.0 cm is present, which invades the right lateral uterine wall. There is associated adjacent pelvic ascites. (d) There is a hyperdense, enhancing lesion in the jejunum in the left hemiabdomen with central hypoattenuation measuring 2.2 × 2.0 × 2.2 cm.

A 48-year-old woman (gravida 1, para 1) with a history of polycystic ovarian syndrome and thyroid cancer treated with radioactive iodine presented to her endocrinologist for follow-up and was found to have an elevated testosterone level. An ultrasound of the pelvis showed enlarged ovaries that were hypoechoic with increased vascularity concerning for a neoplasm (Figures 1(a) and 1(b)). To further evaluate the radiographic findings, the patient was scheduled for laparoscopic bilateral salpingo-oophorectomy (BSO). A complete blood count (CBC) during her presurgical appointment was notable for pancytopenia. The patient was sent for hematology for evaluation. A bone marrow biopsy was performed that showed AML with replacement of the bone marrow by blasts, which by flow cytometry of the bone marrow aspirate were found to express myeloperoxidase (MPO), HLA-DR, dim CD123, CD4, and CD56, but lacked CD34 and CD117. Immunostaining of the bone marrow core biopsy showed similar findings except the blasts were dim MPO-positive and were positive for CD68 (KP1). Cytogenetics showed an abnormal hyperdiploid karyotype with three related clones. Abnormalities included extra copies of chromosome 6, 20, 21, and 22 and included an interstitial deletion of 8q. A myeloid molecular panel, covering 40 genes, showed no mutations. The patient was treated with standard induction chemotherapy (idarubicin and cytarabine). The patient achieved clinical remission, the bone marrow biopsy showed no morphologic or immunophenotypic evidence of residual disease, and cytogenetics showed a normal karyotype. The patient underwent workup for an allogeneic bone marrow transplant, which included, in light of her previous laboratory studies, repeat testing of a serum testosterone level. Her testosterone level was elevated which again led to an ovarian ultrasound that showed findings similar to the first examination. Based on these studies, there was concern for bilateral stromal ovarian tumors. The previously planned laparoscopic BSO was performed. The patient was then admitted to the transplant service to begin transplant conditioning.

**Figure 2:**
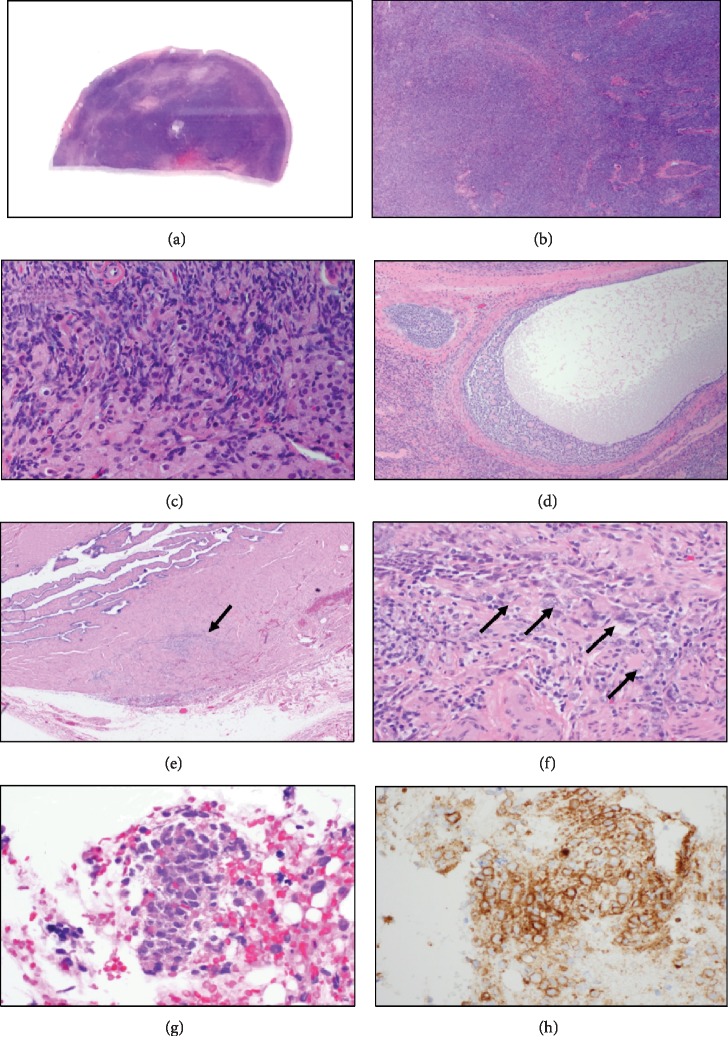
(a) The (right or left) ovary exhibits stromal hyperplasia that replaces the medulla (hematoxylin and eosin). (b) There is a dense proliferation of oval- to spindle-shaped stromal cells in the ovary exhibiting a vaguely nodular pattern (hematoxylin and eosin). (c) There were a few foci of ovarian stromal hyperthecosis consisting of single cells with small clusters of luteinized cells which have abundant, lightly vacuolated cytoplasm (hematoxylin and eosin). (d) A microscopic granulosa cell tumor was seen in the left ovary which showed the microfollicular cell pattern (Call-Exner bodies; hematoxylin and eosin). (e) Cross-section of the fallopian tube showing focal infiltration by atypical, blastic appearing mononuclear cells (arrow; hematoxylin and eosin). (f) The infiltrating blasts had open chromatin and prominent nucleoli. A few with admixed lymphocytes were also present (arrows; hematoxylin and eosin). (g) Endometrial biopsy showing infiltration by the blasts. Note that the blasts have a moderate amount of eosinophilic cytoplasm (hematoxylin and eosin). (h) Immunohistochemical staining showed that the blasts were CD56 positive (immunoperoxidase).

The bilateral ovaries and fallopian tubes showed no abnormalities on gross visual examination. Histologic examination, however, showed an atypical mononuclear cell infiltrate in both fallopian tubes and focally within the left ovary. Immunoperoxidase staining of paraffin tissue sections showed that these mononuclear cells expressed HLA-DR, CD33, CD56, KP1 (CD68), CD123, and dim focal MPO but were negative for CD34 and CD117, an immunophenotypic profile identical to that of the patient's previously diagnosed AML (Figures 2(a)–2(f)). Thus, the findings were consistent with myeloid sarcoma involving the fallopian tubes and ovary. Additional histologic findings showed bilateral ovarian stromal hyperplasia, hyperthecosis, and a microscopic granulosa cell tumor (2 mm), findings consistent with her clinical hyperandrogenism.

Following surgical resection and a pathologic review, the patient's testosterone levels decreased precipitously and a repeat bone marrow biopsy was performed, which showed recurrent AML. The plan for the patient to undergo an allogeneic bone marrow transplant was aborted.

The patient underwent salvage reinduction with high-dose cytarabine and gemtuzumab. A repeat bone marrow biopsy following treatment when the patient was in clinical remission showed no morphologic evidence of disease but showed minimal residual disease based on flow cytometry (0.32% abnormal blasts identified). Approximately two weeks later, the patient began experiencing abnormal uterine bleeding in the setting of worsening thrombocytopenia. Imaging showed an enlarged, heterogeneous uterus with multifocal hypodense and cystic foci (Figure 1(c)). The patient had an endometrial biopsy to determine if this represented leukemic infiltration, a solid tumor malignancy, or other pathology.

The biopsy contained small fragments of endometrium with an atypical mononuclear cell infiltrate. The cells expressed CD56 and HLA-DR, consistent with MS (Figures 2(g) and 2(h)). A concurrent bone marrow biopsy revealed a hypercellular marrow with increased blasts (~10% by immunohistochemistry), consistent with persistent AML. She then reported new-onset hematochezia; a CT scan demonstrated a 2.2 cm enhancing, likely a centrally necrotic lesion in the jejunum representing further extramedullary disease (Figure 1(d)). The patient's clinical status began to rapidly decline with constant rectal and vaginal bleeding. Two months after the diagnosis of relapse, the patient died from cardiopulmonary arrest secondary to disease.

## 3. Discussion

This demonstrates a rare, interesting case of AML diagnosed during the evaluation for known bilateral adnexal masses. Following surgery, she was then found to have myeloid sarcoma of the fallopian tubes and the left ovary with additional findings of bilateral ovarian stromal hyperplasia, hyperthecosis, and a microscopic granulosa cell tumor. Shortly after recurrence of her AML, the patient began experiencing abnormal uterine bleeding in the setting of myeloid sarcoma now with uterine involvement and rapid clinical decline. This case further exemplifies the aggressive nature of myeloid sarcoma after multiple chemotherapy regimens as well as a clear predilection for the gynecologic tract among certain cancer types.

Extramedullary tumors of myeloid cells were first described in the literature by British physician Allan Burns in 1811 [3]. They were later referred to as chloromas by King in 1853, derived from the Greek word *chloros* (green), because of the green-tinged color of these tumors due to the presence of myeloperoxidase (MPO) [4]. Nearly six decades later, Dock and Warthin recognized the connection between “chloromas,” i.e. myeloid sarcomas, and acute leukemia [5]. As these tumors became better understood over time, the term “granulocytic sarcoma,” coined by Rappaport in 1967, was used to describe any extramedullary manifestation of AML [6]. In 2008, the World Health Organization (WHO) classified the definition of MS as “a tumor mass consisting of myeloid blasts with or without maturation occurring at an anatomic site other than the bone marrow.” [7] As per the 2017 update of the WHO Classification of Tumours of Haematopoietic and Lymphoid Tissues, “myeloid sarcoma” is the current correct terminology for these extramedullary myeloid tumors [8].

The pathogenesis of female reproductive organ involvement with MS is not fully understood. Neural cell adhesion molecule (NCAM; CD56), which is present in normal ovarian, testicular, and gastrointestinal tissue, is believed to play a role in the homing of MS to specific tissues [9]. Additionally, reproductive organs have inherent barriers, which are hypothesized to serve as sanctuary sites for leukemic cells to proliferate despite systemic therapy [10]. Literature has shown that once a single focus of extramedullary disease is identified, progression in other organs as well as the bone marrow usually follows within a year [11]. Furthermore, this case shows that complete bone marrow response may not signify that sanctuary sites, like the ovaries, are free of disease.

The clinical and pathologic diagnosis of MS has been challenging historically. MS most commonly occurs concurrently with AML with an estimated incidence between 2.5 and 9.1% of patients [12]. However, primary (isolated) MS occurring outside of AML is exceedingly rare with reported rates as low as two cases per million adults. Isolated MS can differ from concurrent disease as regards tumor behavior, effectiveness of treatment, and prognosis. Given the diversity of MS presentations and locations, as well as low clinical suspicion, this disease commonly goes misdiagnosed [12]. Meis et al. reported a misdiagnosis rate of MS as high as 75% in a 1986 retrospective series [13]. More recent reports now demonstrate that nearly 25-50% of all patients with MS will go misdiagnosed [12, 14]. The most common reason cited for misdiagnoses has been inadequate immunophenotyping of the MS lesion [15]. Immunohistochemical staining is important in the correct diagnosis, as MS lesions express both myeloid and monocytoid antigens including CD45, CD43, and CD15 [16].

There has been scarce literature investigating the survival and prognosis of patients with MS. First-line treatment usually involves systemic induction chemotherapy as surgical excision does not delay spread of disease or improve prognosis [16, 17]. There have also been case reports that suggest that a robust inflammatory response following a surgical procedure could worsen MS or lead to a postoperative persistent febrile syndrome [18].

MS most commonly manifests in the skin, subcutaneous tissues, and the lymph nodes. Claerhout et al. conducted a case series of 41 patients with de novo MS and 31 patients with secondary disease [19]. Among both cohorts, respectively, they found that MS localized to the skin in 24-25.5% of cases, lymph nodes (18.2-16.0%), gastrointestinal tract [20] (10.9-2.0%), breast (7.3-10.0%), ovary (3.6-0.0%), cervix/urine corpus (1.8-4.0%), bone (3.6-6.0%), and brain (3.6-6.0%). There have also been reports of MS invading into the vulva. Although MS of the gynecologic tract is exceedingly rare, it is hypothesized that it may actually be underdiagnosed based on postmortem studies showing frequent gynecologic involvement in women who have died of myeloid leukemia [21, 22].

This case report is consistent with previous rare literature reports and adds to the current literature. One of the largest previous reported series of MS involving the gynecologic tract, Garcia et al. reported a series of 11 cases at a single center case series from 2006 spanning the previous 31 years [2]. Among gynecologic organs, the uterus was the most frequent site of disease—involved in 8 patients. Myeloid sarcoma was confined to the ovary in 2 patients, involved the fallopian tubes in 2 patients, and was confined to the clitoris in 1 patient. Four of the patients exhibited concurrent involvement of multiple gynecologic sites. Other studies have described cases with multisite involvement in the gynecologic tract, including one with disease in the cervix, mesosalpinx, and ovaries. In addition, cases with disease concurrently involving the gynecologic tract and extragynecologic sites have also been reported [22].

## 4. Conclusion

In conclusion, myeloid sarcoma of the gynecologic tract represents an important and often misdiagnosed hematologic malignancy in an underrecognized site. As this paper has demonstrated, MS in the setting of AML and extramedullary relapse is exceedingly rare; however, it likely occurs more than previously understood, especially given the postmortem findings and persistence in preserved ovarian tissue. Physicians should have an increased awareness regarding this potential site of disease with a high index of suspicion. As immunophenotyping is the primary cause of misdiagnosis, appropriate tissue with immunohistochemical staining is important. Additional studies are necessary to better understand this rare manifestation, and in turn allowing for better understanding of myeloid malignancies in general as well.

## References

[1] Avni B., Koren-Michowitz M. (2011). Myeloid sarcoma: current approach and therapeutic options. *Therapeutic Advances in Hematology*.

[2] Garcia M. G., Deavers M. T., Knoblock R. J. (2006). Myeloid sarcoma involving the gynecologic tract: a report of 11 cases and review of the literature. *American Journal of Clinical Pathology*.

[3] Burns A. (1811). *Observations of Surgical Anatomy in Head and Neck*.

[4] King A. (1853). Case of Chloroma. *Monthly Journal of Medical Science*.

[5] Dock G., Warthin A. (1904). A new case of chloroma with leukemia. *Transactions of the Association of American Physicians*.

[6] Rappaport H. (1967). *Tumors of the Hematopoietic System. In: Atlas of Tumor Pathology, Section III, Fascicle 8*.

[7] Swerdlow S. H., Campo E., Harris N. L. (2008). *WHO Classification of Tumours of Haematopoietic and Lymphoid Tissues*.

[8] Pileri S. A., Orazi A., Falini B., Swerdlow S. H., Capo E., Harris N. L. (2017). *Myeloid Sarcoma in WHO Classification of Tumours of Haematopoietic and Lymphoid Tissue*.

[9] Byrd J. (1994). Recurrent granulocytic sarcoma. An unusual variation of acute myelogenous leukemia associated with 8;21 chromosomal translocation and blast expression of the neural cell adhesion molecule. *Cancer*.

[10] Bakst R. L., Tallman M. S., Douer D., Yahalom J. (2011). How I treat extramedullary acute myeloid leukemia. *Blood*.

[11] Chong G., Byrnes G., Szer J., Grigg A. (2000). Extramedullary relapse after allogeneic bone marrow transplantation for haematological malignancy. *Bone Marrow Transplantation*.

[12] Almond L. M., Charalampakis M., Ford S. J., Gourevitch D., Desai A. (2017). Myeloid sarcoma: presentation, diagnosis, and treatment. *Clinical Lymphoma, Myeloma & Leukemia*.

[13] Meis J. M., Butler J. J., Osborne B. M., Manning J. T. (1986). Granulocytic sarcoma in nonleukemic patients. *Cancer*.

[14] Seifert R. P., Bulkeley W., Zhang L., Menes M., Bui M. M. (2014). A practical approach to diagnose soft tissue myeloid sarcoma preceding or coinciding with acute myeloid leukemia. *Annals of Diagnostic Pathology*.

[15] Wilson C. S., Medeiros L. J. (2015). Extramedullary manifestations of myeloid neoplasms. *American Journal of Clinical Pathology*.

[16] Capote S., Sánchez-Iglesias J. L., Cubo-Abert M. (2018). Myeloid sarcoma as a simulator of advanced ovarian cancer: a case report. *European Journal of Obstetrics, Gynecology, and Reproductive Biology*.

[17] Wang P., Li Q., Zhang L., Ji H., Zhang C. Z., Wang B. (2017). A myeloid sarcoma involving the small intestine, kidneys, mesentery, and mesenteric lymph nodes: a case report and literature review. *Medicine*.

[18] Tripathi R., Sharma B., Chaturvedi K. U., Khurana N., Mala Y. M. (2005). Granulocytic sarcoma of the female genital tract: report of a case with an unusual presentation. *Gynecologic and Obstetric Investigation*.

[19] Claerhout H., van Aelst S., Melis C. (2018). Clinicopathological characteristics of de novo and secondary myeloid sarcoma: a monocentric retrospective study. *European Journal of Haematology*.

[20] Nazer A., Al-Badawi I., Chebbo W., Chaudhri N., El-Gohary G. (2012). Myeloid sarcoma of the vulva post-bone marrow transplant presenting as isolated extramedullary relapse in a patient with acute myeloid leukemia. *Hematology/Oncology and Stem Cell Therapy*.

[21] Lucia S. P., Mills H., Lowenhaupt E., Hunt M. L. (1952). Visceral involvement in primary neoplastic diseases of the reticulo-endothelial system. *Cancer*.

[22] Hernandez J. A., Navarro J.-T., Rozman M. (2002). Primary myeloid sarcoma of the gynecologic tract: a report of two cases progressing to acute myeloid leukemia. *Leukemia & Lymphoma*.

